# The discovery of Cas9's *trans*‐cleavage activity: Unlocking new molecular diagnostic tools

**DOI:** 10.1002/ctm2.70384

**Published:** 2025-06-19

**Authors:** Ying Chen, Jiyun Chen, Liang Liu

**Affiliations:** ^1^ State Key Laboratory of Cellular Stress Biology, Department of Biochemistry, School of Life Sciences, Faculty of Medicine and Life Sciences, Xiamen University Xiamen Fujian China

**Keywords:** Cas9, *Trans*‐cleavage

Rapid and accurate nucleic acid testing is of vital importance in clinical treatment for disease prevention, diagnosis and prognosis judgment.^1^ Since the type I,^2^ V^3^ and VI^4^ CRISPR‐Cas effector nucleases showed *trans*‐cleavage activity for non‐specific nucleic acids after target binding, researchers have developed a series of efficient nucleic acid detection tools, which can achieve high sensitivity and specificity in pathogen detection.^3^, ^5^, ^6^ Recently, our research innovatively revealed that the type II CRISPR‐Cas9 system possesses *trans*‐cleavage activity with multiple target activation and multiple cutting substrates.^7^ By combining the *trans*‐cleavage activity of Cas9 with nucleic acid amplification technology, we successfully developed a new nucleic acid detection platform, expanding the application of the CRISPR–Cas9 system from gene editing to nucleic acid‐based diagnostics.

## 1 CRISPR‐BASED DIAGNOSTICS

In recent years, molecular diagnostic techniques based on the CRISPR–Cas system have made breakthrough progress due to their unique nucleic acid targeting and cutting activity. Research reports that the effector protein Cas13a of the type VI CRISPR–Cas system, activates the *trans*‐cleavage activity towards non‐specific single‐stranded RNA (ssRNA) after specifically recognizing the target RNA.^5^ Similarly, the effector proteins of the type V CRISPR–Cas system also exhibit the *trans*‐cleavage ability towards non‐specific single‐stranded DNA (ssDNA) upon target DNA binding.^3^

By combining this characteristic with isothermal amplification technology, researchers have successfully developed a variety of nucleic acid detection platforms, including SHERLOCK and DETECTR.^3^, ^5^, ^8^ These breakthrough technologies effectively overcomethe dependence of traditional PCR methods on profssional equipment and shorten detection time, demonstrating significant advantages in portability and reaction speed. However, existing systems still have room for improvement in detection sensitivity, multiplex detection capabilities and clinical validation. To further promote the development of molecular diagnostic technologies and achieve true point‐of‐care (POC) testing, it is particularly important to develop more CRISPR–Cas systems with application potential.^5^

## 2 Cas9 POSSESSES crRNA–tracrRNA DIRECTED *trans*‐CLEAVAGE ACTIVITY

However, developing technologies that can achieve POC detection faces multiple key challenges. First, the existing amplification‐free detection techniques are difficult to achieve clinical‐grade detection sensitivity while ensuring rapid response. Second, mainstream CRISPR detection technologies (such as the Cas12 and Cas13 systems) have significant barriers to technological transformation, which directly leads to high costs of diagnostic reagents and limited technological iterations. To break through this predicament, we shifted our research focus on the type II CRISPR–Cas9 systems. Although this system dominates the field of gene editing with its precise *cis*‐cutting target double‐stranded DNA (dsDNA) activity, its application potential in nucleic acid detection has long been overlooked.^9^

**FIGURE 1:**
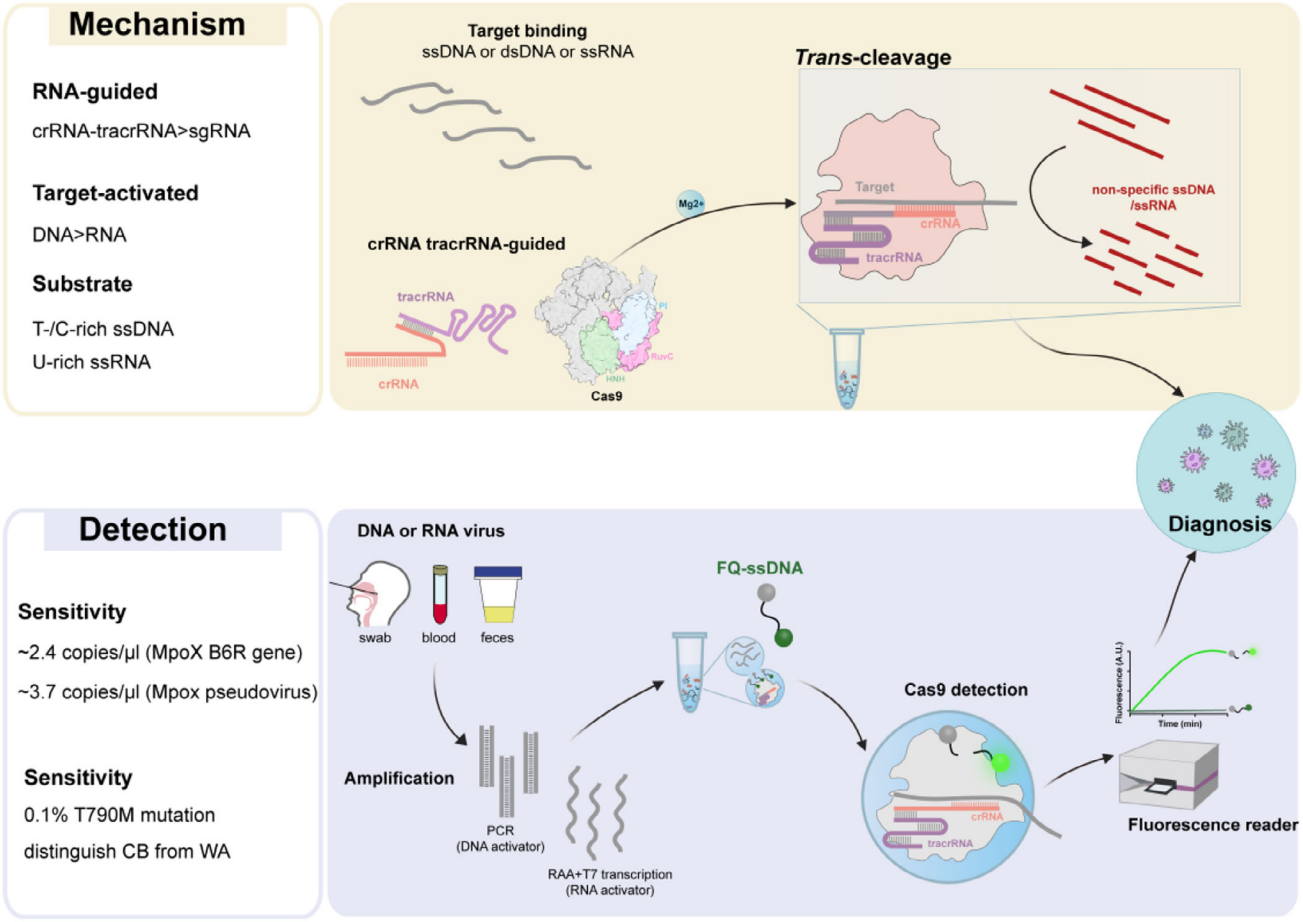
Schematic representation of Cas9's *trans*‐cleavage activity and the DACD and RACD assay. Figure adapted from Ref. ^7^.

**FIGURE 2:**
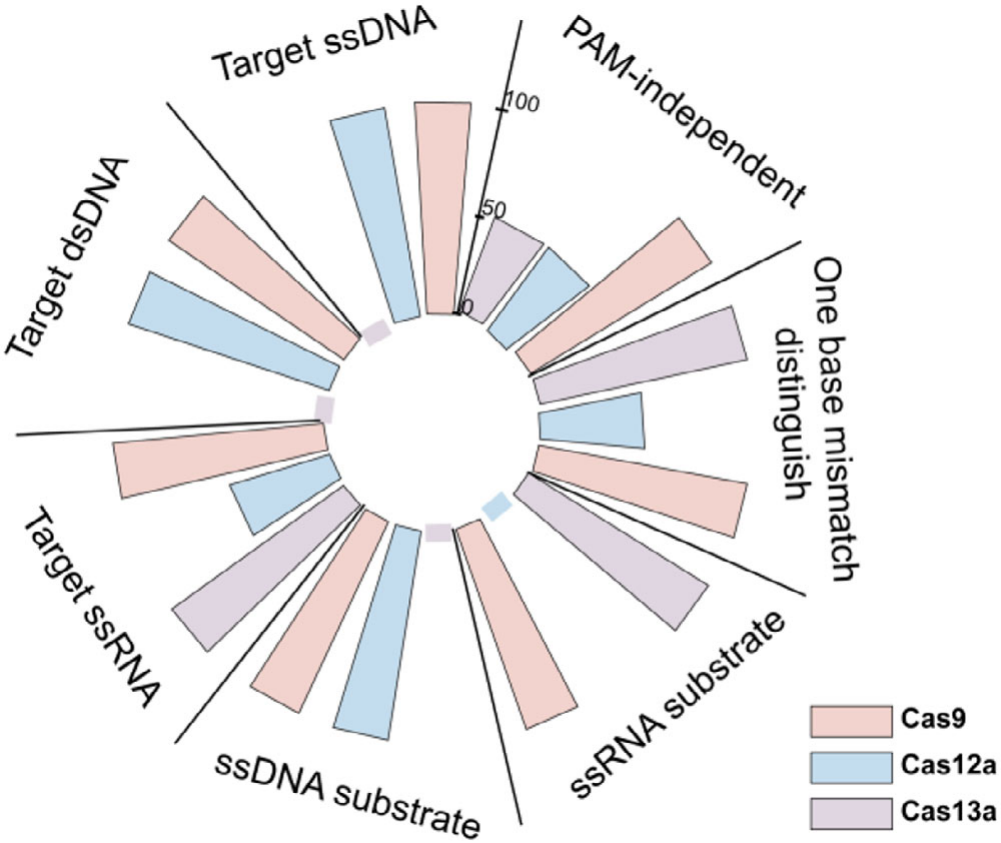
Comparison of *trans*‐cutting characteristics in the CRISPR–Cas systems. The radial bar chart compared the characteristics related to the *trans*‐cutting activity of Cas9 (pink), Cas12a (blue) and Cas13a (purple) in nucleic acid detection. Figure adapted from Ref. ^7^.

By systematically evaluating the *trans*‐cleavage activity of Cas9 protein on sequence‐ and structure‐specific nucleic acid substrates, we innovatively discovered that Cas9 shows *trans*‐cleavage preference for ssDNA substrates rich in T or C under RNA guidance, see Figure 1.^7^ This discovery suggests that *trans*‐cleavage activity may be a broad‐spectrum cleavage mechanism of the CRISPR–Cas system. It is worth noting that when the natural crRNA and *trans*‐activating crRNA (tracrRNA) dual RNAs was used to replace chimeric sgRNA, the catalytic efficiency of Cas9's *trans*‐cleavage was significantly increased by 6–95 times.^7^ Its more efficient *trans*‐cutting activity provides a brand‐new molecular tool for the development of CRISPR‐based nucleic acid detection technology. Furthermore, we used target ssDNA, dsDNA and ssRNA to investigate the conditions for the *trans*‐cleavage activity of Cas9.^7^ The results showed that both DNA and RNA targets could activate the *trans*‐cleavage ability of Cas9 on non‐specific ssDNA or ssRNA substrates.^7^ This dual advantages of multi‐target activation and multiple substrates make Cas9 exhibit unique application potential in the field of nucleic acid detection, see Figure 2. First, its multi‐target response capability can significantly expand the target molecule recognition range of the detection system and reduce its dependence on PAM or PFS sequences during the detection process. Second, the characteristic of multiple cutting substrates provides the possibility of constructing diversified signal amplification systems.

## 3 THE NOVEL *trans*‐CLEAVAGE ACTIVITY OF Cas9 OPENS UP A NEW DIRECTION FOR NUCLEIC ACID DETECTION APPLICATIONS

Our previous results indicated that Cas9 shows *trans*‐cutting activity, but it is still unclear whether this activity would cause accidents in Cas9‐based gene editing applications. Our immune‐protection assays directly discussed this issue, the results showed that the *trans‐*ssDNA cleavage activity of Cas9 could not help *Escherichia coli* resist the infection of M13 bacteriophage.^7^ It is worth noting that by exploring the nuclease domains dependent on the *trans*‐cutting activity of Cas9, we found that the *cis*‐cut of non‐complementary DNA strand dependent on the RuvC domain has the same preference as the *trans*‐cut of ssDNA substrate,^7^ providing an optimal solution for the selection of target sequences in the gene editing process of Cas9.

By combining the *trans*‐cleavage activity of Cas9 with nucleic acid amplification technology, we have developed nucleic acid detection platforms with independent intellectual property rights–DNA‐activated Cas9 detection (DACD) and RNA‐activated Cas9 detection (RACD), which can achieve highly sensitive and specific detection of various viruses and tumor resistance mutations(Figure 1).^7^ Compared with Cas12a and Cas13a, although SpyCas9 has a higher catalytic efficiency than FnCas12a and LshCas13a, it is lower than LbCas12a and LwaCas13a.^7^ However, when we measured the limits of detection (LoD) of Cas9 for target DNA and RNA without amplification, these data were comparable to those of the reported Cas12 and Cas13 systems.^7^ Furthermore, by optimising the *trans*‐cutting system (including buffers, pH, different metal ions, temperature and metal ion concentrations),^7^ the *trans*‐cleavage activity of Cas9 can be significantly enhanced, indicating that the *trans*‐cleavage efficiency of Cas9 has great optimisation space and development potential.

## 4 FUTURE OUTLOOK

The breakthrough discovery of the novel *trans*‐cleavage activity of the type II CRISPR–Cas9 system not only expands the application boundaries of CRISPR–Cas technology, but also promotes the development of molecular diagnosis, basic research and clinical medicine. In the future, we will further optimise the detection technology based on *trans*‐cleavage activity of Cas9 to achieve POC diagnosis for clinical samples.

## AUTHOR CONTRIBUTION

Ying Chen wrote the manuscript and drew the figures. Jiyun Chen and Liang Liu provided valuable discussion and revised the manuscript. All authors have read and approved the article.

## CONFLICT OF INTEREST STATEMENT

The authors declare no conflict of interest.

## ETHICS STATEMENT

Not applicable.
